# Supplementary material for: An Assessment of the Climate Change Impacts on the Distribution of the Glacial Relict Woodpecker Three-Toed Woodpecker Picoides tridactylus
Source: Animals (Basel). 2024 Jun 26;14(13):1879. doi: 10.3390/ani14131879 (PMC11240539; doi:10.3390/ani14131879)
Supplement: Supplementary file 1 [file animals-14-01879-s001.zip › animals-3026212-supplementary.pdf]

Table S1. Percent contribution and permutation importance of environmental variables in all 20 models.

Climatic scenarios	Percent contribution (%)	Permutation importance (%)
Current	BIO10 (31.5)	BIO10 (56.3)
	CLC (26.2)	BIO18 (18.6)
	elevation (21.8)	CLC (11.5)
	BIO18 (16.6)	BIO9 (5.1)
	BIO9 (2.2)	BIO8 (3.8)
	slope (0.8)	slope (3)
	BIO8 (0.7)	elevation (1.4)
	aspect (0.2)	aspect (0.2)
Last interglacial	BIO10 (81.8)	BIO10 (78)
	BIO18 (11.2)	BIO9 (8.7)
	BIO9 (3.5)	BIO18 (8.3)
	BIO8 (3.4)	BIO8 (5.1)
Last glacial maximum MIROC-ESM	BIO10 (81.2)	BIO10 (76.7)
	BIO18 (12.8)	BIO18 (10.3)
	BIO8 (3.4)	BIO9 (8.5)
	BIO9 (2.6)	BIO8 (4.5)
Last glacial maximum CCSM4	BIO10 (80.8)	BIO10 (78)
	BIO18 (12.1)	BIO18 (9.7)
	BIO9 (3.5)	BIO9 (7.8)
	BIO8 (3.5)	BIO8 (4.6)
HadGEM3-GC31-LL 2050 SSP2-4.5	BIO10 (80.6)	BIO10 (74.8)
	BIO18 (12)	BIO9 (9.8)
	BIO8 (3.8)	BIO18 (9.3)
	BIO9 (3.6)	BIO8 (6)
HadGEM3-GC31-LL 2070 SSP2-4.5	BIO10 (81.3)	BIO10 (75.1)
	BIO18 (11.6)	BIO9 (10.2)
	BIO9 (3.6)	BIO18 (9.9)
	BIO8 (3.6)	BIO8 (4.7)
HadGEM3-GC31-LL 2050 SSP5-8.5	BIO10 (81.1)	BIO10 (75.5)
	BIO18 (11.9)	BIO18 (10.6)
	BIO9 (3.6)	BIO9 (8.7)
	BIO8 (3.4)	BIO8 (5.2)
HadGEM3-GC31-LL 2070 SSP5-8.5	BIO10 (81.3)	BIO10 (75.3)
	BIO18 (11.6)	BIO9 (10.7)
	BIO9 (3.7)	BIO18 (9.2)
	BIO8 (3.4)	BIO8 (4.8)
IPSL-CM6A-LR 2050 SSP2-4.5	BIO10 (81.1)	BIO10 (77.6)
	BIO18 (12.2)	BIO18 (9.3)
	BIO8 (3.5)	BIO9 (8.1)
	BIO9 (3.2)	BIO8 (5)
IPSL-CM6A-LR 2070 SSP2-4.5	BIO10 (79.6)	BIO10 (73.2)
	BIO18 (12.5)	BIO18 (10.7)
	BIO9 (4.3)	BIO9 (10.2)
	BIO8 (3.6)	BIO8 (6)
IPSL-CM6A-LR 2050 SSP5-8.5	BIO10 (81)	BIO10 (75.7)
	BIO18 (11.5)	BIO9 (10.4)
	BIO8 (3.9)	BIO18 (8.1)
	BIO9 (3.6)	BIO8 (5.8)

IPSL-CM6A-LR 2070 SSP5-8.5	BIO10 (79.5)<br>BIO18 (12.9)<br>BIO9 (3.9)<br>BIO8 (3.7)	BIO10 (72.5)<br>BIO18 (12)<br>BIO9 (9.4)<br>BIO8 (6.1)
MRI-ESM2-0 2050 SSP2-4.5	BIO10 (79.9)<br>BIO18 (12.5)<br>BIO9 (4.2)<br>BIO8 (3.4)	BIO10 (76.1)<br>BIO18 (9.6)<br>BIO9 (9.2)<br>BIO8 (5.1)
MRI-ESM2-0 2070 SSP2-4.5	BIO10 (80.2)<br>BIO18 (12.7)<br>BIO9 (3.6)<br>BIO8 (3.5)	BIO10 (79)<br>BIO18 (8.9)<br>BIO9 (7.5)<br>BIO8 (4.6)
MRI-ESM2-0 2050 SSP5-8.5	BIO10 (81.1)<br>BIO18 (10.9)<br>BIO9 (4.5)<br>BIO8 (3.5)	BIO10 (77.7)<br>BIO9 (9.6)<br>BIO18 (7.8)<br>BIO8 (4.8)
MRI-ESM2-0 2070 SSP5-8.5	BIO10 (80.5)<br>BIO18 (13.4)<br>BIO8 (3.1)<br>BIO9 (3)	BIO10 (74.8)<br>BIO18 (11.8)<br>BIO9 (8.7)<br>BIO8 (4.7)
MIROC6 2050 SSP2-4.5	BIO10 (80.2)<br>BIO18 (12.6)<br>BIO9 (3.9)<br>BIO8 (3.3)	BIO10 (76.1)<br>BIO18 (9.7)<br>BIO9 (9.1)<br>BIO8 (5.1)
MIROC6 2070 SSP2-4.5	BIO10 (82.2)<br>BIO18 (11)<br>BIO9 (3.6)<br>BIO8 (3.2)	BIO10 (77.7)<br>BIO9 (8.8)<br>BIO18 (8.5)<br>BIO8 (5)
MIROC6 2050 SSP5-8.5	BIO10 (80.8)<br>BIO18 (12.2)<br>BIO9 (3.6)<br>BIO8 (3.4)	BIO10 (74.8)<br>BIO9 (10.8)<br>BIO18 (8.9)<br>BIO8 (5.6)
MIROC6 2070 SSP5-8.5	BIO10 (80.6)<br>BIO18 (12.4)<br>BIO9 (3.5)<br>BIO8 (3.5)	BIO10 (72.3)<br>BIO18 (12)<br>BIO9 (10.2)<br>BIO8 (5.5)

Note. BIO8 is mean temperature of the wettest quarter, BIO9 is mean temperature of the driest quarter, BIO10 is mean temperature of the warmest quarter, and BIO18 is precipitation of the warmest quarter.

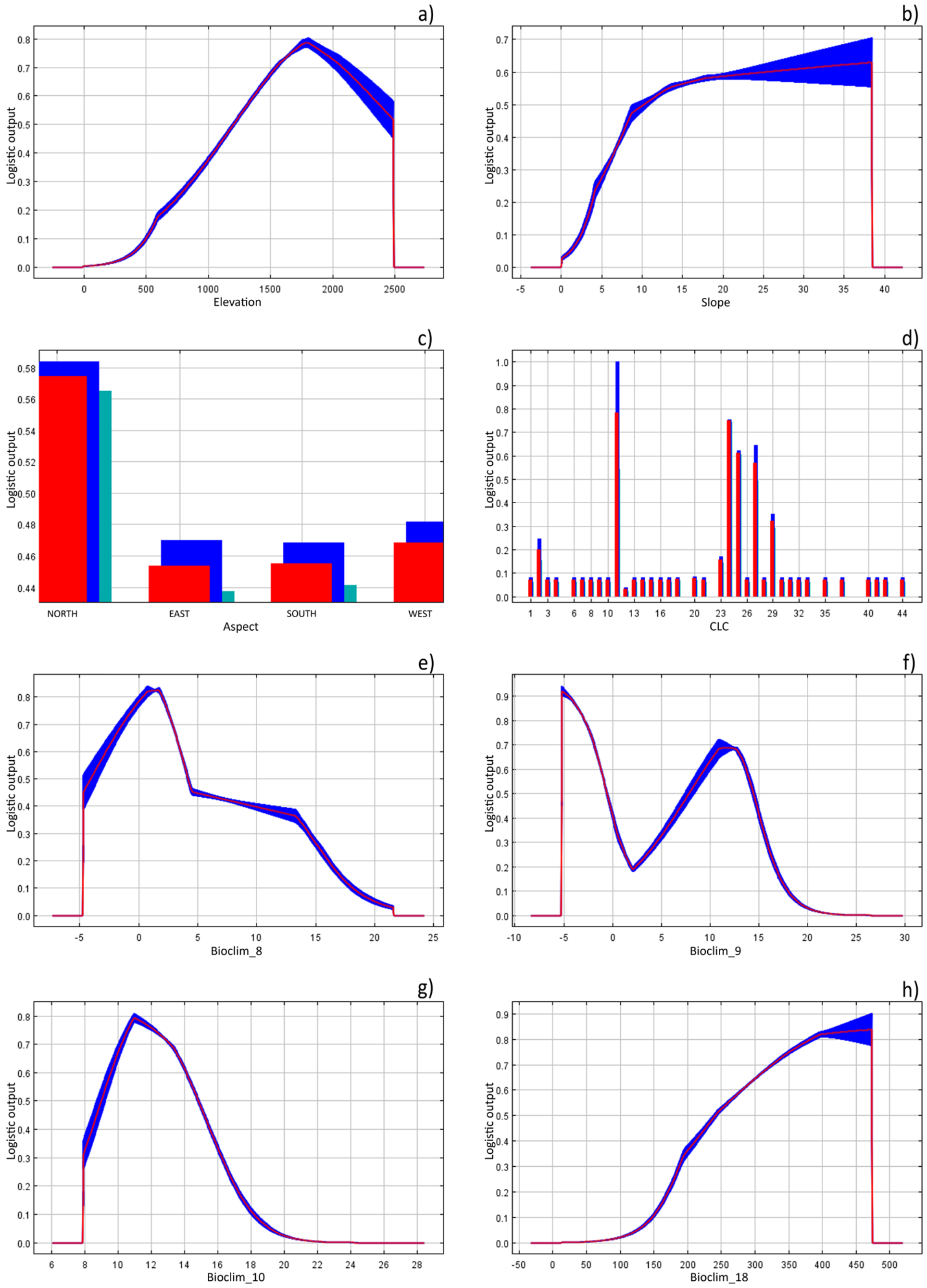

Figure S1. Response curves of environmental variables used to create the distribution model of Three-Toed Woodpecker. (**a**) Elevation; (**b**) Slope; (**c**) Aspect; (**d**) CLC; (**e**) Mean temperature of the wettest quarter; (**f**) Mean temperature of the driest quarter; (**g**) Mean temperature of the warmest quarter; (**h**) Precipitation of the warmest quarter. The range of values of the environmental variables is on the x-axis, whereas habitat suitability, represented through the logistic output, is on the y-axis. Red shows the mean value for 10 replicate runs, and blue shows the standard deviation.

Definition of the CLC classes on the x-axis: (**1**) Continuous urban fabric; (**2**) Discontinuous urban fabric; (**3**) Industrial or commercial units; (**4**) Road and rail networks and associated land; (**6**) Airports; (**7**) Mineral extraction sites; (**8**) Dump sites; (**9**) Construction sites; (**10**) Green urban areas; (**11**) Sport and leisure facilities; (**12**) Non-irrigated arable land; (**13**) Permanently irrigated land; (**14**) Rice fields; (**15**) Vineyards; (**16**) Fruit trees and berry plantations; (**17**) Olive groves; (**18**) Pastures; (**20**) Complex cultivation patterns; (**21**) Land principally occupied by agriculture, with significant areas of natural vegetation; (**23**) Broad-leaved forest; (**24**) Coniferous forest; (**25**) Mixed forest; (**26**) Natural grassland; (**27**) Moors and heathland; (**28**) Sclerophyllous vegetation; (**29**) Transitional woodland-shrub; (**30**) Beaches, dunes, sands; (**31**) Bare rocks; (**32**) Sparsely vegetated areas; (**33**) Burnt areas; (**35**) Inland marshes; (**37**) Salt marshes; (**40**) Water courses; (**41**) Water bodies; (**42**) Coastal lagoons; (**44**) Sea and ocean.

Table S2. Area of three categories of suitable habitats (km^2) for Three-Toed Woodpecker in the present, past (last interglacial and last glacial maximum), and future (average 2050 and average 2070 in two climate scenarios, SSP2-4.5 and SSP5-8.5), as well as the percentage of area change (%) concerning the current period.

**Climatic scenarios**	**Poorly suitable habitat**		**Moderately suitable habitat**		**Highly suitable habitat**		**Total**	
	**Area (km^2)**	**% change**	**Area (km^2)**	**% change**	**Area (km^2)**	**% change**	**Area (km^2)**	**% change**
Current	26,217	–	5,257	–	375	–	31,849	–
Last interglacial	247	−99.06	10	−99.81	0	−100	257	−99.19
Last glacial maximum MIROC-ESM	97,360.01	271.36	44,183.99	740.48	65,518.01	17,371.47	207,062.01	550.14
Last glacial maximum CCSM4	123,527	371.17	21,158	302.47	6,904	1,741.07	151,589	375.96
Average LGM	110,443.51	321.27	32,671	521.48	36,211.01	9,556.27	179,325.51	463.05
HadGEM3-GC31-LL 2050 SSP2-4.5	4,474	−82.93	84	−98.40	0	−100	4,558	−85.69
HadGEM3-GC31-LL 2070 SSP2-4.5	4,725	−81.98	341	−93.51	1	−99.73	5,067	−84.09
HadGEM3-GC31-LL 2050 SSP5-8.5	2,381	−90.92	69	−98.69	0	−100	2,450	−92.31
HadGEM3-GC31-LL 2070 SSP5-8.5	54	−99.79	0	−100	0	−100	54	−99.83
IPSL-CM6A-LR 2050 SSP2-4.5	5,501	−79.02	143	−97.28	12	−96.8	5,656	−82.24
IPSL-CM6A-LR 2070 SSP2-4.5	3,262	−87.56	137	−97.39	14	−96.27	3,413	−89.28
IPSL-CM6A-LR 2050 SSP5-8.5	2,517	−90.40	45	−99.14	7	−98.13	2,569	−91.93
IPSL-CM6A-LR 2070 SSP5-8.5	154	−99.41	0	−100	0	−100	154	−99.52
MRI-ESM2-0 2050 SSP2-4.5	10,867	−58.55	1,204	−77.10	70	−81.33	12,141	−61.88
MRI-ESM2-0 2070 SSP2-4.5	8,424	−67.87	595	−88.68	95	−74.67	9,114	−71.38
MRI-ESM2-0 2050 SSP5-8.5	7,594	−71.03	588	−88.81	57	−84.8	8,239	−74.13
MRI-ESM2-0 2070 SSP5-8.5	3,787	−85.56	57	−98.92	5	−98.67	3,849	−87.91
MIROC6 2050 SSP2-4.5	7,904	−69.85	396	−92.47	60	−84	8,360	−73.75
MIROC6 2070 SSP2-4.5	2,785	−89.38	188	−96.42	0	−100	2,973	−90.67
MIROC6 2050 SSP5-8.5	3,696	−85.90	73	−98.61	19	−94.93	3,788	−88.11
MIROC6 2070 SSP5-8.5	556	−97.88	5	−99.90	0	−100	561	−98.24
Average 2050 SSP2-4.5	7,186.5	−72.59	456.75	−91.31	35.5	−90.53	7,678.75	−75.89
Average 2070 SSP2-4.5	4,799	−81.70	315.25	−94	27.5	−92.67	5,141.75	−83.86

Average 2050 SSP5-8.5	4,047	−84.56	193.75	−96.31	20.75	−94.47	4,261.5	−86.62
Average 2070 SSP5-8.5	1,137.75	−95.66	15.5	−99.71	1.25	−99.67	1,154.5	−96.38
